# Performance characterization and biocompatibility assessment of silicone polyurethanes for polymer heart valve applications

**DOI:** 10.1039/d4ra00183d

**Published:** 2024-04-03

**Authors:** Bixuan Liu, Zhihua Liu, Haiyang Wei, Yana Meng, Qianwen Hou, Aili Wang, Yongkai Zhang, Enhui Han, Shengshou Hu, Jianye Zhou

**Affiliations:** a State Key Laboratory of Cardiovascular Disease, Fuwai Hospital, National Center for Cardiovascular Diseases, Chinese Academy of Medical Sciences and Peking Union Medical College Beijing 100037 China bixuanliu@163.com 18729950840@163.com 346284746@qq.com hqw@fuwaihospital.org wangaili96@163.com yk790039@gmail.com 2811664802@qq.com huss@fuwaihospital.org zhoujianye@fuwaihospital.org; b School of Materials Science and Engineering, Beijing Institute of Technology Beijing 100081 China 1346816911@qq.com

## Abstract

Silicone polyurethanes have gained widespread application in the biomedical field due to their excellent biocompatibility. This study comprehensively investigates four silicone polyurethane materials suitable for polymer heart valves, each exhibiting distinct chemical compositions and structural characteristics, leading to significant differences, particularly in mechanical performance and biocompatibility. Surface analysis reveals an elevated surface silicon element content in all materials compared to the bulk, indicating a migration of silicon elements towards the surface, providing a structural basis for enhancing biological stability and biocompatibility. However, higher silicon content leads to a decrease in mechanical performance, potentially resulting in mechanical failure and rupture in artificial heart valves. Concerning biocompatibility, an increase in silicone content diminishes the material's adsorption capability for cells and proteins, consequently improving its biocompatibility and biological stability. In summary, while high silicone content leads to a reduction in mechanical performance, the formation of a “silicon protective layer” on the material surface mitigates cell and protein adsorption, thereby enhancing biocompatibility and biological stability. Through comprehensive testing of the four silicone polyurethane materials, this study aims to provide insightful perspectives and methods for selecting materials suitable for polymer heart valves. Additionally, the thorough performance exploration of these materials serves as a crucial reference for the performance assessment and biocompatibility research of polymeric artificial heart valve materials.

## Abstract 2

Silicone polyurethanes have gained widespread application in the biomedical field due to their excellent biocompatibility.

## Graphical abstract

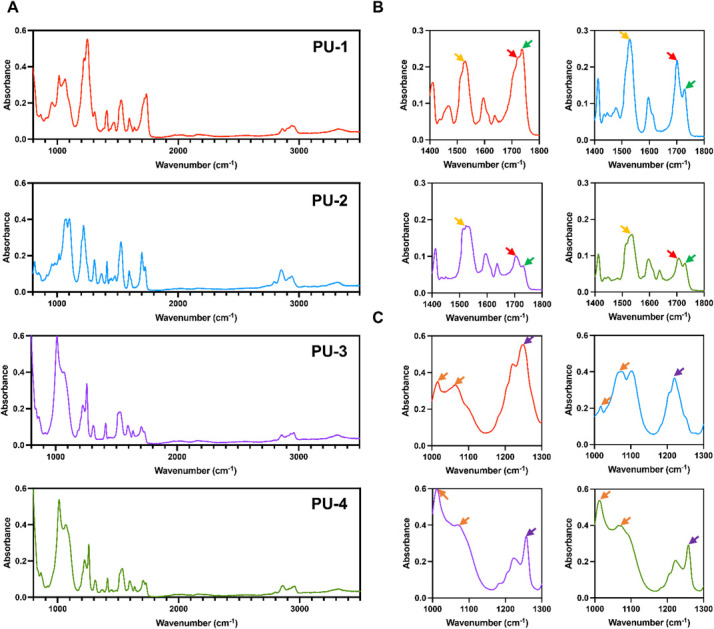

## 1 Introduction

Heart valve diseases fall under the category of structural heart diseases, significantly impacting the quality of life for patients and posing potential life-threatening risks.^1^ With the continuous increase in the aging population, the number of patients with valve diseases is on the rise,^2^ becoming a major medical challenge in the cardiovascular field. Consequently, there is a growing urgency for the development of artificial heart valves at present.

Currently, clinically utilized artificial heart valves are primarily divided into two categories: mechanical valves and biological valves.^3,4^ Mechanical valves, widely applied due to their outstanding durability, suffer from poor hemodynamics owing to their rigid characteristics, leading to thrombus formation and necessitating lifelong anticoagulant therapy for patients.^5,6^ In contrast, biological valves exhibit superior hemodynamic performance and excellent biocompatibility; however, their durability is relatively poor,^7^ prone to collagen degradation under physiological conditions, resulting in pathological changes such as calcification, valve thickening, and tearing.^8^ Given these limitations in both types of artificial heart valves, there is an urgent need for the development of a new generation of artificial heart valves that combine both biocompatibility and biological durability to better meet the demands of disease treatment.

In recent years, significant progress has been made in the field of heart valve materials, with a valve material based on silicone polyurethane urea demonstrating excellent mechanical performance and biocompatibility,^9–12^ providing feasibility for the development of a new generation of artificial heart valves. As the application of polymer materials in heart valve technology continues to advance, finding polymers with optimal biocompatibility and biological stability has become one of the most challenging frontiers in the biomedical field.^13,14^ Simultaneously, the preparation, properties, and application research of biomedical polymer materials have garnered considerable attention from the academic and industrial sectors.^15–17^ However, the development and application of polymer materials for artificial heart valves remain a highly challenging issue. It requires attention not only to the material's basic properties such as microstructure, surface morphology10, and hydrophilicity/hydrophobicity^18^ but also to its mechanical performance^19^ to ensure its ability to withstand the mechanical environment inside the heart valve and other applications. More importantly, as polymer heart valves serve as long-term implant materials, emphasis must be placed on biocompatibility, biological stability, and durability.^20^

This study comprehensively characterizes the performance and assesses the biocompatibility of four silicone polyurethane materials. Through testing methods such as SAXS and DSC, the microphase separation structure of the materials is explored. Additionally, analysis of the material's surface/interface properties is conducted using XPS, SEM-EDX, and mechanical performance testing. This helps gain in-depth insights into the structure and performance of silicone polyurethane materials with different structures, providing a foundational performance data basis for selecting high-polymer materials suitable for artificial heart valves. Moreover, we conducted cytotoxicity experiments, inflammation reaction studies, analysis of blood cell adhesion and activation, protein adsorption experiments, and subcutaneous implantation experiments in rats, comprehensively evaluating the biocompatibility and biological stability of these materials to provide a more comprehensive assessment of their feasibility in biomedical applications.

In conclusion, as polymer materials continue to be widely applied in the field of heart valves, this study aims to explore in-depth the performance characteristics and biocompatibility of different silicone polyurethane materials. It provides valuable references for the future development of materials for heart valve repair and replacement, with the potential to positively impact the field of biomedical science.

## 2 Experimental

### 2.1 Materials

PU-1, PU-3, PU-4 were siliconized polyurethane materials provided by Beijing Institute of Technology, and PU-2 was provided by Hubei University. Polyurethane 80 A was purchased from Lubrizol Corporation in the United States (Table 1).

**Table 1:** Composition, molecular weights and molecular weight distribution of PUs

Material	Type	Structural composition		*M*_n_ (kg mol^−1^)	PDI	Source
		Soft segment	Hard segment			
PU-1	Siloxane polycarbonate polyurethane	PCDL/PDMS	MDI/BDO	84	1.8	BIT^a^
PU-2	Siloxane polyether polyurethane	PHMO/PDMS	MDI/BDO	51	1.5	HBU^b^
PU-3	Siloxane polyether polyurethane	PHMO/PDMS	MDI/BHTD/BDO	92	2.1	BIT^a^
PU-4	Siloxane polyether polyurethane	PTMG/PDMS	MDI/EDA/BDO	103	2	BIT^a^
80 A	Polyether polyurethane	PTMG	MDI/BDO	98	1.7	Lubrizo

a BIT: School of Materials Science and Engineering, Beijing Institute of Technology.

b HBU: Hubei University.

### 2.2 Methods

#### 2.2.1 Fourier transform infrared spectroscopy (FTIR)

Thermo Scientific Nicolet IS10 FTIR was used to measure the infrared spectra of the samples with a thickness of approximately 0.3 mm. The scanning parameters were set to 30 scans and a resolution of 4 cm^−1^, covering the range of 500–4000 cm^−1^

#### 2.2.2 Scanning electron microscopy and energy dispersive X-ray spectroscopy (SEM-EDX)

High-resolution field emission scanning electron microscopy (CAMECA SXFiveFE, France) was used to capture images of the samples in secondary electron mode. Energy-dispersive X-ray spectroscopy (EDX) analysis was performed to determine the elemental distribution on the material surfaces. Images were obtained at 5 kV with a working distance of 4.0 mm, and all samples were carbon-coated before testing for improved conductivity.

#### 2.2.3 X-ray photoelectron spectroscopy (XPS) analysis

XPS was employed to analyze the elemental composition of the samples and characterize their surface properties. The instrument used was Phi Quantera-IISXM (UlvacPhi, Japan) with an AlKα X-ray source (Al target, 1486.6 eV, linewidth 0.68 eV). Different depth analyses (0–200 nm) were performed after etching.

#### 2.2.4 Thermogravimetric analysis (TGA)

The thermal stability of siliconized polyurethane materials was assessed using a TG-DTA 8122 thermogravimetric analyzer (Rigaku, Japan). Testing conditions included an air atmosphere, a temperature range of 25 °C to 600 °C, and a heating rate of 10 °C min^−1^.

#### 2.2.5 Differential scanning calorimetry (DSC)

DSC analysis was conducted to determine the glass transition temperature (*T*_g_) of siliconized polyurethane materials. The instrument used was a DSC 200 F3 differential scanning calorimeter (Netzsch, Germany). The testing conditions involved heating from −150 °C to 200 °C at a rate of 10 °C min^−1^, followed by cooling to −150 °C at the same rate, all under ambient air.

#### 2.2.6 Small-angle X-ray scattering (SAXS) experiment

SAXS experiments were performed using the Xeuss 2.0 instrument from Xenocs (France). Samples were placed on the sample holder, and the instrument parameters were set with a copper target, a power of 30 W, a wavelength of 1.54189 Å, and a Pilatus 3R 300K detector with a single pixel size of 172 μm.

#### 2.2.7 Mechanical properties

Sample preparation involved cutting specimens into dumbbell shapes using a precision cutter. Tensile tests were performed on the prepared samples using a UTM6102 electronic universal testing machine (China) with a stretching speed of 100 mm min^−1^. Creep and recovery experiments were conducted using the same instrument, applying a constant stress of 4 MPa for 60 minutes and allowing a 30 minutes recovery period.

#### 2.2.8 Wetting angle and water absorption analysis

Contact angle measurements were performed at room temperature using the JC2000C1 static drop contact angle/interface tension meter (Shanghai, China). To measure water absorption, 1 cm × 1 cm samples with thicknesses ranging from 0.2 mm to 0.4 mm were cut and dried in a 60 °C oven until a constant mass (*W*_0_) was achieved. Afterward, the samples were immersed in double-distilled water at 37 °C for 96 hours, dried, and weighed again (*W*_1_). The water absorption was calculated using the formula: water absorption = (*W*_1_ − *W*_0_)/*W*_0_.

#### 2.2.9 Cell toxicity

##### 2.2.9.1 Preparation of materials and extracts

Materials were first placed in a 75% ethanol solution for 3 hours to disinfect (as the materials were insoluble in ethanol). Subsequently, the materials were washed three times with sterile PBS to remove residual ethanol. The materials were then placed under a UV lamp for 30 minutes to ensure thorough sterilization. Extracts were prepared by immersing the sterilized materials in DMEM culture medium containing 10% fetal bovine serum for 24 hours at 37 °C.

##### 2.2.9.2 Cell toxicity assay

The L929 mouse fibroblast cell line was used for the cell toxicity assay. The cells were cultured in DMEM medium containing 10% fetal bovine serum at 37 °C in a 5% CO_2_ incubator. Experimental groups included extracts from four siliconized polyurethane polymers, blank control group (DMEM culture medium with 10% fetal bovine serum), negative control group (extract from biocompatible material 80 A), and positive control group (extract from a polymer with high tin content). After 24 hours of cell seeding, 100 μL of each extract was added to the wells, and the cells were cultured for an additional 3 days.

Cell Counting Kit-8 (CCK-8) assay was performed to evaluate cell viability. After removing the culture medium, a mixture of 100 μL DMEM and 10 μL CCK-8 reagent was added to each well, followed by incubation for 2 hours at 37 °C. Absorbance was measured at 450 nm using a microplate reader. The relative viability was calculated as: relative viability = (absorbance of the experimental group − absorbance of the blank control group)/(absorbance of the positive control group − absorbance of the blank control group).

##### 2.2.9.3 Cell morphology observation

For cell morphology observation, L929 mouse fibroblasts and HUVEC human umbilical vein endothelial cells were selected. The cells were cultured in conditions similar to those described earlier. After 24 hours, the original culture medium was replaced with 1 mL of the respective extract, and the cells were cultured for an additional 3 days. Cells were stained with May–Grunwald and Giemsa stains, and cell morphology was observed under an inverted fluorescence microscope.

#### 2.2.10 Macrophage adhesion and inflammatory response

##### 2.2.10.1 Macrophage adhesion

The Ana-1 mouse macrophage cell line was used for the experiment. These cells were cultured in RPMI-1640 medium containing 10% fetal bovine serum and maintained at 37 °C in a 5% CO_2_ incubator. Materials were cut into 1 cm × 1 cm squares, disinfected thoroughly, and placed in 24-well plates. Ana-1 cells were seeded at a density of 15 000 cells per well and cultured for 3 days. Afterward, the samples were processed for staining, and macrophage adhesion was observed using a fluorescence microscope.

##### 2.2.10.2 Inflammatory response

After co-culturing macrophages with materials for 3 and 7 days, cell culture supernatants were collected, and ELISA kits were used to quantitatively analyze the secretion of the cytokines G-CSF and IL-10 by macrophages, assessing the inflammatory response induced by the materials.

#### 2.2.11 Adhesion and activation of blood cells

##### 2.2.11.1 Adhesion of blood cells

Materials were cut into 1 cm × 1 cm squares and placed in 15 mL centrifuge tubes. Fresh whole blood from healthy volunteers (3 mL per tube) was added, and the tubes were incubated at 37 °C with shaking for 24 hours to promote interaction between blood cells and material surfaces. After incubation, the materials were removed from the tubes, washed three times with PBS to remove non-adherent blood cells, fixed in 2.5% glutaraldehyde solution, and prepared for electron microscopy. Images were captured using a microscope at 500× magnification.

##### 2.2.11.2 Lactate dehydrogenase (LDH) semi-quantitative assay

Materials were cut into 1 cm × 1 cm squares and placed in 5 mL centrifuge tubes. Fresh whole blood from healthy volunteers (1 mL per tube) was added, and the tubes were incubated at 37 °C with shaking for 3 and 24 hours. After incubation, the materials were removed, washed three times with PBS, and placed in 24-well plates. Then, 1 mL of 0.05% Triton X-100 was added to each well, and the plates were incubated for 5 minutes to fully lyse the cells. A semi-quantitative analysis of lactate dehydrogenase (LDH) released from blood cells was performed using an LDH assay kit.

##### 2.2.11.3 Platelet activation

Materials (1 cm × 1 cm) were placed in 24-well plates, and 1 mL of platelet-rich plasma (PRP) was added to each well. After incubation at 37 °C for 60 minutes, simulating the interaction between materials and plasma in the body, the plasma was collected, and the β-thromboglobulin (β-TG) content in the supernatant was determined using an ELISA kit. The data were statistically analyzed to assess the ability of the tested materials to activate platelets.

##### 2.2.11.4 Coagulation four tests

Blood was collected from healthy volunteers using sodium citrate (1 : 9 anticoagulant ratio). After standing at 4 °C for 30 minutes, the blood samples were centrifuged at 3500 rpm at 4 °C for 10 minutes to obtain plasma. Materials cut into 1 cm × 1 cm squares were placed in tubes, and 1 mL of plasma was added to each tube. The tubes were incubated at 37 °C for 60 minutes, ensuring thorough mixing and reaction. Coagulation four tests, including activated partial thromboplastin time (APTT), prothrombin time (PT), fibrinogen (FIB), and thrombin time (TT), were analyzed using a semi-automatic blood coagulation analyzer (Beijing Saikexide).

#### 2.2.12 Protein adsorption test

Materials were incubated in 1 mg mL^−1^ BSA solution and 0.1 mg mL^−1^ FBG solution at 37 °C for 12 hours on a constant temperature shaker to allow proteins to fully contact the material surface. After incubation, the materials were rinsed three times with PBS to remove unabsorbed proteins. The materials were then placed in a 1% SDS solution, shaken thoroughly to dissolve the proteins adhered to the material surface, and the protein content was determined using a BCA assay kit.

#### 2.2.13 Subcutaneous implantation experiment in rats

##### 2.2.13.1 Subcutaneous implantation

The animal experimental protocol was approved by the Institutional Animal Care and Use Committee (IACUC), Fuwai Hospital, Chinese Academy of Medical Sciences (Ethics No: FW-2022-0038), and complied with the principles of laboratory animal care. Two-week-old rats weighing around 100 g were selected for the experiment. After anesthesia, sample pieces cut into 1 cm × 1 cm size were implanted into the dorsal area of each rat. Each rat was implanted with 4 sample pieces, and each sample had 8 replicates.

##### 2.2.13.2 Hematoxylin and eosin (HE) staining

After 30 days of implantation, rats were euthanized with carbon dioxide, and the implanted samples were collected. The samples were placed in a solution containing 10% formaldehyde for fixation. After fixation, the samples were dehydrated using a gradient ethanol process, embedded in paraffin, and sliced into 5 μm sections for subsequent staining and histological analysis. Finally, the sections were placed on glass slides, and HE staining was performed.

##### 2.2.13.3 The determination of calcium content by ICP-OES method

In terms of sample handling, we placed material slices extracted from the subcutaneous tissue of rats in a constant temperature oven at 80 °C until a constant weight was achieved. Following sample preparation, quantitative analysis of calcium elements was conducted using an ICP-OES instrument (PerkinElmer Optima 8000, USA). The samples were initially subjected to digestion treatment before being introduced into the ICP instrument for measurement. Throughout the measurement process, a series of standard calcium solutions were utilized for instrument calibration. All procedures were carried out in accordance with international standard methods to ensure the reliability and accuracy of the measurement results.

## 3 Results

### 3.1 Performance characterization

For polymer heart valves, the polymer material used as a long-term implant must possess biostability, biocompatibility, and mechanical properties. Due to its unique location, higher demands are placed on overall performance, particularly exceptional biostability and comprehensive mechanical properties, such as an implantation life of up to 15 years or more, low creep, moderate modulus, fatigue resistance, and excellent tensile performance.^21,22^ Accurate and comprehensive characterization of material performance is crucial not only for material selection but also for guiding material design and synthesis. This can truly achieve the goal of using molecular structure design to achieve desired performance. The polymer materials' performance characterization testing methods in this study include structural analysis, interfacial analysis, microphase separation analysis, thermal stability analysis, and mechanical performance analysis. Through these tests, we aim to establish connections between biocompatibility, biostability, and performance, providing essential data support for material selection, design, and medical applications.

#### 3.1.1 Structural analysis

**Fig. 1:**
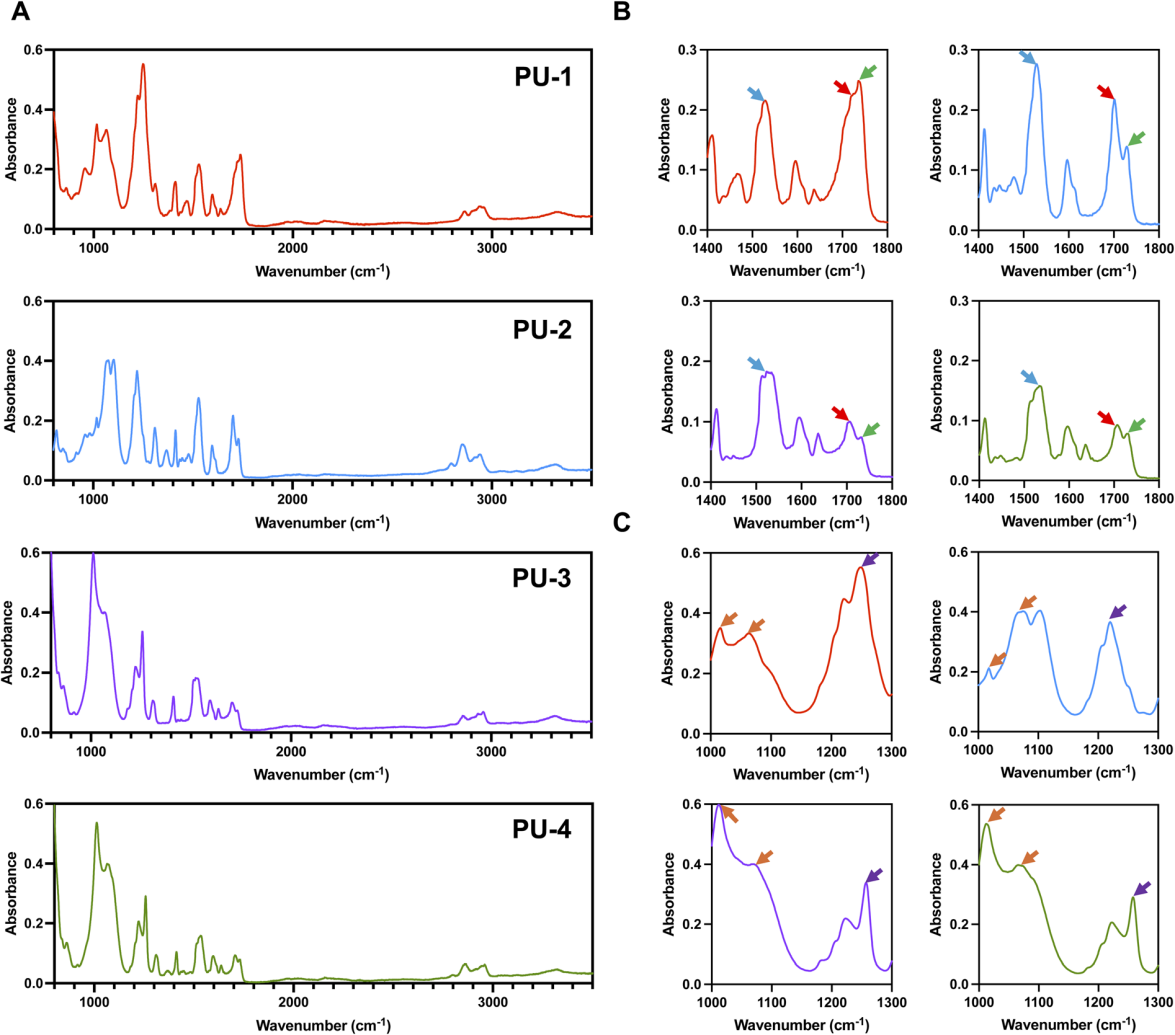
Structural analysis of PUs. (A) FTIR absorption spectra of four silicone–oxygen polyurethanes. (B) Characteristic absorption peaks of polyurethane, with yellow arrows indicating N–H and C–N, red arrows indicating hydrogen-bonded C=O, and green arrows indicating non-hydrogen-bonded C=O. (C) Characteristic absorption peaks of silicone–oxygen, with orange arrows indicating Si–O–Si and purple arrows indicating CH_2_–Si (CH_3_).

Siloxane polyurethane is typically synthesized using polydimethylsiloxane diol as the soft segment, or partially as the soft segment, through the polymerization of diisocyanate and small-molecule chain extenders. According to literature reports9,^23–25^, the characteristic absorption peaks of polyurethane are at 1710 cm^−1^ (hydrogen-bonded C=O), 1735 cm^−1^ (non-hydrogen-bonded C=O), and 1530 cm^−1^ (N–H and C–N). The characteristic absorption peaks of siloxane are around 1020 cm^−1^ and 1080 cm^−1^ (Si–O–Si). Siloxane molecules are linked to the soft segment of polyurethane through the CH_2_–Si(CH_3_) bond (1260 cm^−1^). As shown in Fig. 1, the mentioned infrared characteristic absorption peaks are evident in all four polymer materials, indicating that they are all siloxane polyurethanes.

#### 3.1.2 Interfacial analysis

**Fig. 2:**
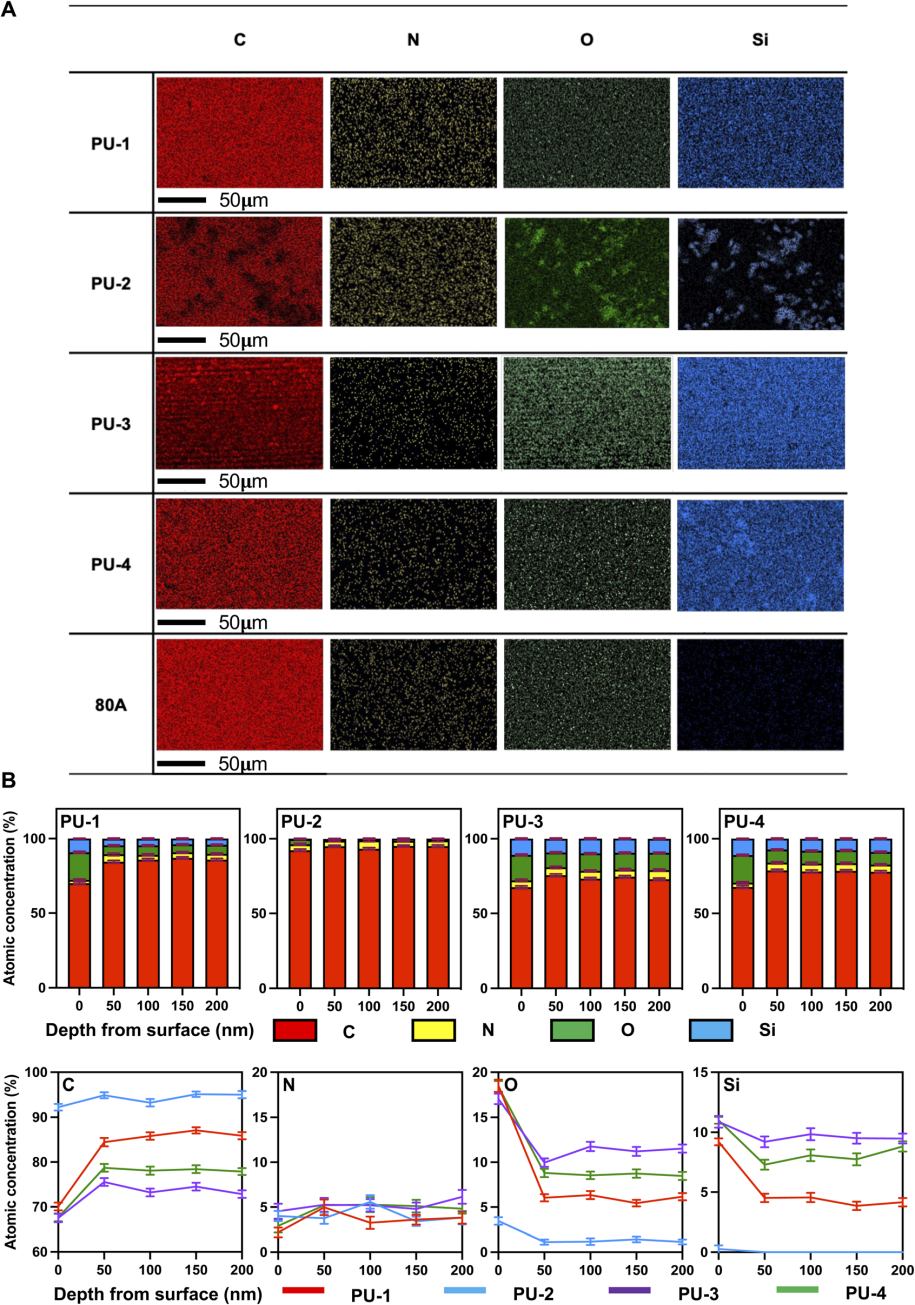
Interfacial analysis of PUs. (A) SEM-EDX layered images for C (red), N (yellow), O (green) and Si (blue) mapping of PUs at the surface. (B) Compositional depth profiles of atomic concentrations of C, N, O, and Si in PUs verses etch depth (0–200 nm).

To determine the elemental composition of the material surface, we first used scanning electron microscopy combined with energy-dispersive X-ray spectroscopy (SEM-EDX) to measure the contents of C, N, O, and Si elements on the material surface. Fig. 2 shows the elemental content on the surfaces of the four siloxane polyurethanes and polyurethane 80 A. PU-1, PU-2, PU-3, and PU-4 all contain silicon, with uniform distribution in PU-1, PU-3, and PU-4, and higher silicon content in PU-3 and PU-4. PU-2 exhibits uneven silicon distribution and lower silicon content. Polyurethane 80 A does not contain silicon. Based on SEM-EDX analysis, we further employed X-ray photoelectron spectroscopy (XPS) to study the element distribution on the materials' surfaces in-depth. We demonstrate the distribution of C, N, O, and Si elements during the depth etching process. The *X*-axis indicates the relative distance from the surface with increasing etching depth. The results indicate a significant enrichment of silicon at the material surface. Further analysis reveals a gradual decrease in silicon concentration with increasing etching depth, showing a clear decreasing trend.

#### 3.1.3 Microphase separation analysis

**Fig. 3:**
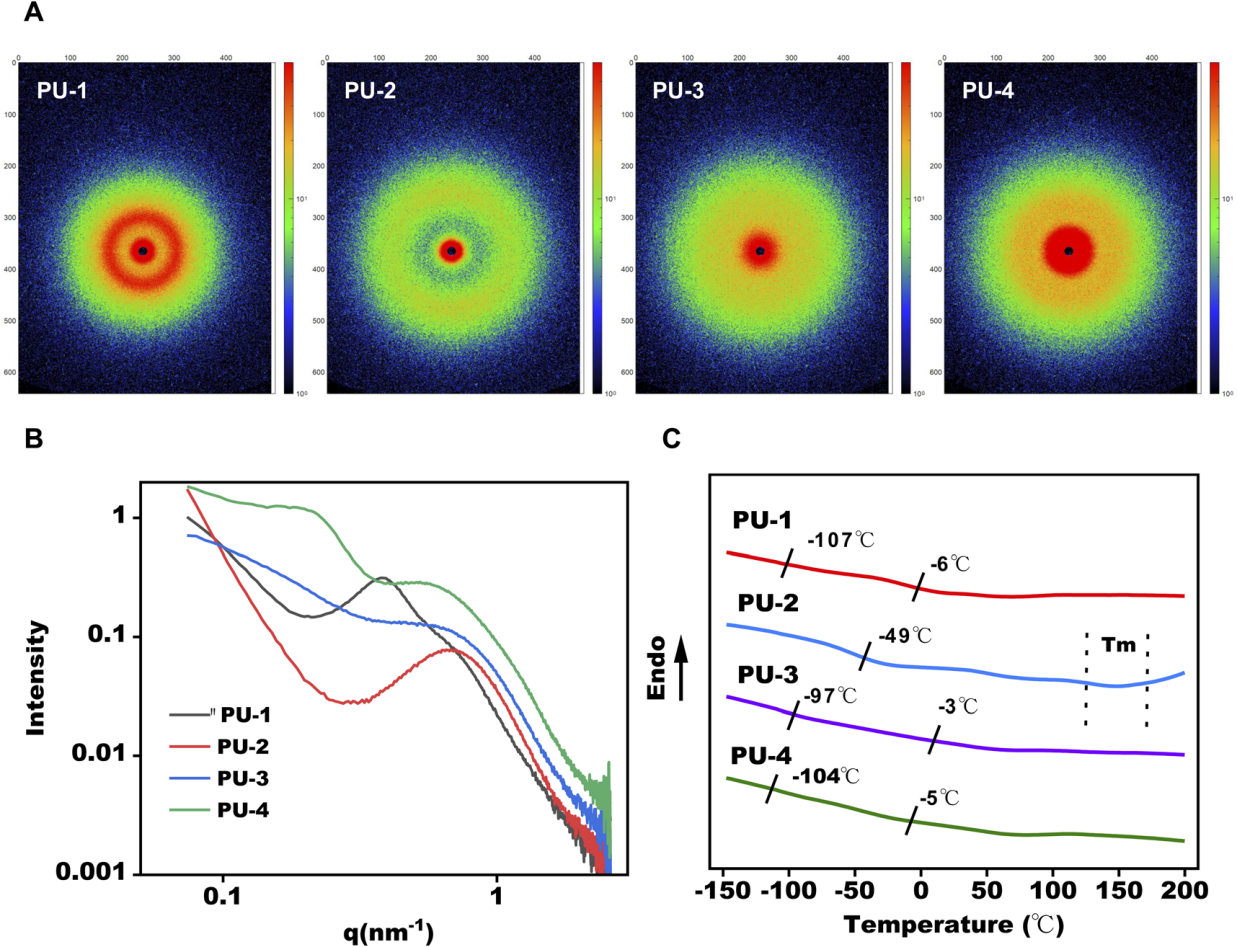
Microphase separation analysis of PUs. (A and B) Small angle X-ray scattering (SAXS) patterns for PUs. (C) DSC thermograms of PUs.

The nano-sized structure of polyurethane can be observed using X-ray diffraction, with small-angle X-ray scattering (SAXS) being effective in characterizing the microstructure of polyurethane by scattering electrons to obtain the distribution of electron density in the material. The microphase separation degree of siloxane-containing polyurethane can be obtained using Bragg's law sin *θ* = *λ*/*d* and the scattering angle formula *q* = 4π sin *θ*/*λ*. All siloxane-containing polyurethanes exhibit clear first-order scattering peaks in their SAXS scattering intensity distribution curves, indicating the presence of microphase separation structures in polyurethane samples. Using the mentioned equations, the calculated phase interval distances for these samples are all within the range of 15–30 nm. As shown in Fig. 3, the peak values of *q* in the SAXS spectra, closer to *q* = 0, indicate a greater degree of microphase separation. This suggests that PU-1 and PU-4 exhibit a more noticeable microphase separation degree, while PU-2 and PU-3 have a lower microphase separation degree. All samples' SAXS two-dimensional scattering patterns show circular scattering rings, indicative of the typical features of microphase separation between soft and hard segments and suggesting a random orientation of these microphase regions.

Through differential scanning calorimetry (DSC) testing, we observe two glass transition temperatures in PU-1: −107 °C corresponds to the glass transition of polydimethylsiloxane (PDMS), and −6 °C is the glass transition temperature of polycarbonate. PU-2 does not show the *T*_g_ of PDMS, likely due to its lower silicon content and lower degree of phase separation. PU-3 and PU-4 both exhibit two glass transition temperatures, with PU-3's −97 °C and −3 °C corresponding to the glass transitions of polydimethylsiloxane and polyether, respectively. PU-4's −104 °C and −5 °C correspond to the glass transitions of polydimethylsiloxane and polyether, respectively. Furthermore, PU-3's glass transition temperature of polydimethylsiloxane at −97 °C is significantly lower than that of PU-1 and PU-4, indicating a lower degree of microphase separation in PU-3, consistent with SAXS results.

#### 3.1.4 Thermal stability of materials

**Fig. 4:**
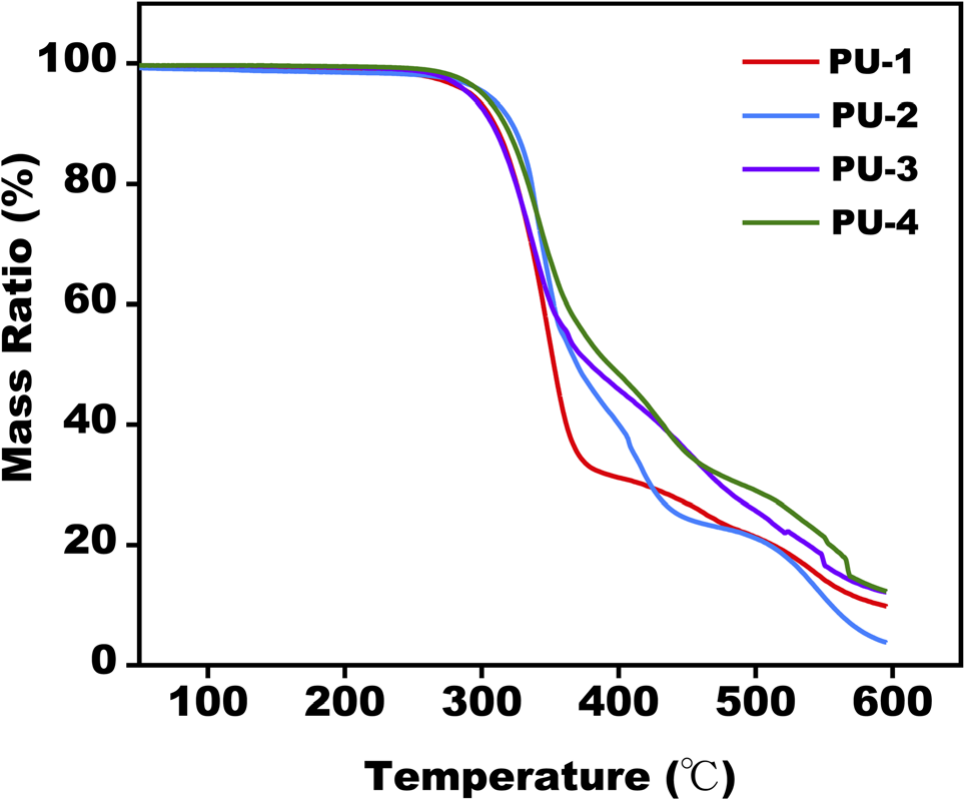
TGA weight loss behavior for PUs.

Fig. 4 displays the curves of thermal weight loss with temperature for siloxane polyurethane materials. The results show that all materials start thermal degradation around 270 °C, and the maximum decomposition temperature is after 300 °C, conforming to the general thermal degradation pattern of polyurethane materials, demonstrating good thermal stability. Moreover, it is noted that after reaching 600 °C, the residual rates of the materials follow the order: PU-3 ≈ PU-4>PU-1>PU-2, consistent with the sequence of silicon addition obtained from surface analysis.

#### 3.1.5 Mechanical performance

**Fig. 5:**
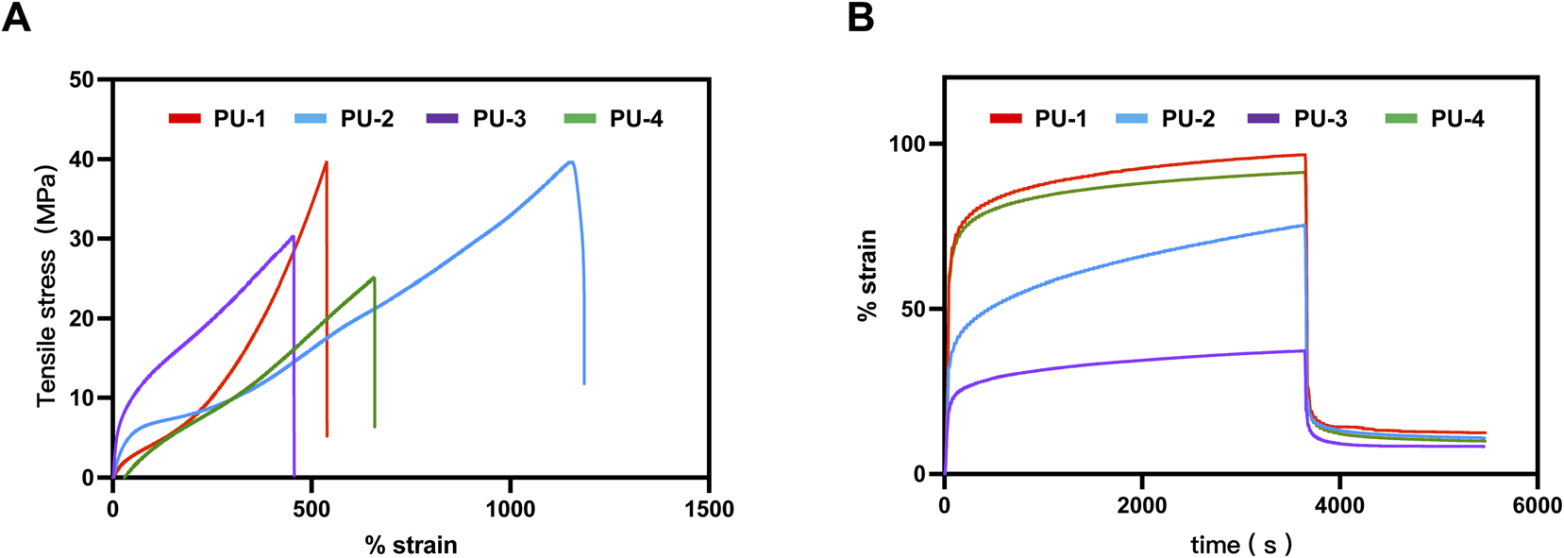
Microphase separation analysis of PUs. (A) Stress–strain curves of PUs. (B) Creep and recovery strain-time curves of PUs.

Materials for polymer heart valves must have excellent mechanical properties, including suitable dynamic modulus to ensure normal hemodynamics and valve opening and closing, high tensile strength, high elongation at break, low creep, and fatigue resistance. Fig. 5 shows the axial tensile performance and creep recovery performance of the four siloxane polyurethanes. PU-1 and PU-2 exhibit better mechanical performance, with maximum tensile strength exceeding 30 MPa (Table 2), while PU-3 and PU-4, due to their high silicon content, show slightly inferior mechanical performance compared to PU-1 and PU-2.

**Table 2:** Tensile properties of PUs

SiPUU	Elongation at break (%)	Ultimate tensile stress (MPa)	Young's modulus (MPa)
PU-1	561.42 ± 23.82	36.31 ± 3.43	10.74 ± 0.93
PU-2	1165.61 ± 22.04	37.67 ± 1.51	16.83 ± 0.92
PU-3	464.38 ± 10.33	29.71 ± 1.09	34.85 ± 2.55
PU-4	616.51 ± 12.04	25.47 ± 0.30	10.05 ± 1.48

#### 3.1.6 Wettability and water absorption

**Fig. 6:**
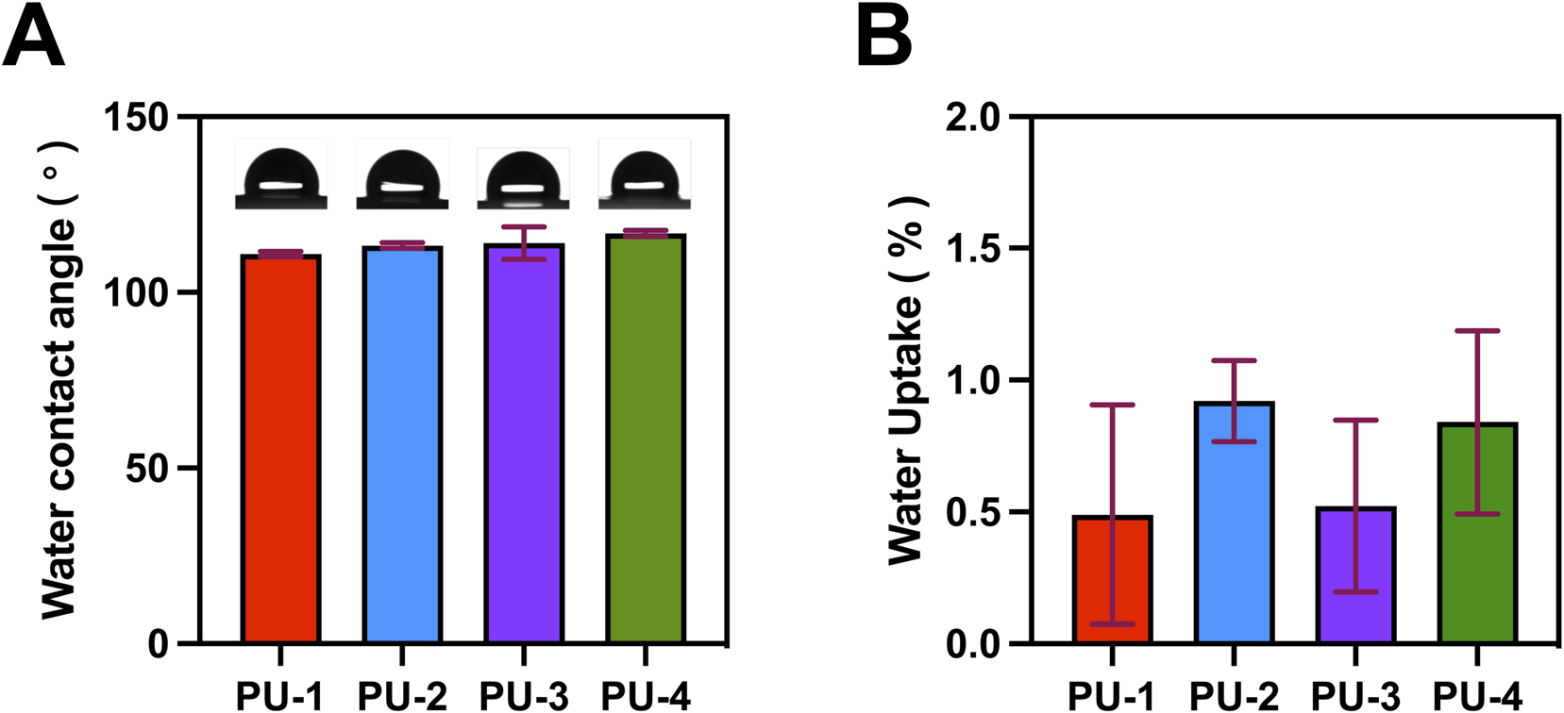
Wettability and water absorption of PUs. (A) Water contact angle of PUs. (B) Water absorption of PUs.

Through contact angle experiments, we assessed the surface hydrophobicity of the materials. The results indicate that the contact angles of all materials are greater than 90°, suggesting they are hydrophobic materials. This characteristic is crucial for medical devices that come into contact with blood, such as heart valves, as hydrophobic surfaces help reduce the contact between the material and biological fluids, thereby slowing down material degradation and enhancing stability and lifespan. Furthermore, we conducted a detailed analysis of the water absorption rates for each material, as presented in Fig. 6. The water absorption rates for all materials remain below 2%, with PU-1 and PU-3 showing slightly lower water absorption rates compared to the other two materials. This indicates that these two materials exhibit slightly lower liquid absorption capacity in humid environments, which is crucial for maintaining material stability and performance.

### 3.2 Biocompatibility

The design and evaluation of medical biomaterials require careful consideration of biocompatibility to ensure safety in clinical applications. This entails avoiding inflammation, cytotoxicity, and tissue damage caused by the biomaterial. In this study, we assessed the biocompatibility of four siloxane polyurethanes from several aspects, including cytotoxicity, inflammatory response, and the material's degradation level under *in vivo* conditions.

#### 3.2.1 Cytotoxicity

**Fig. 7:**
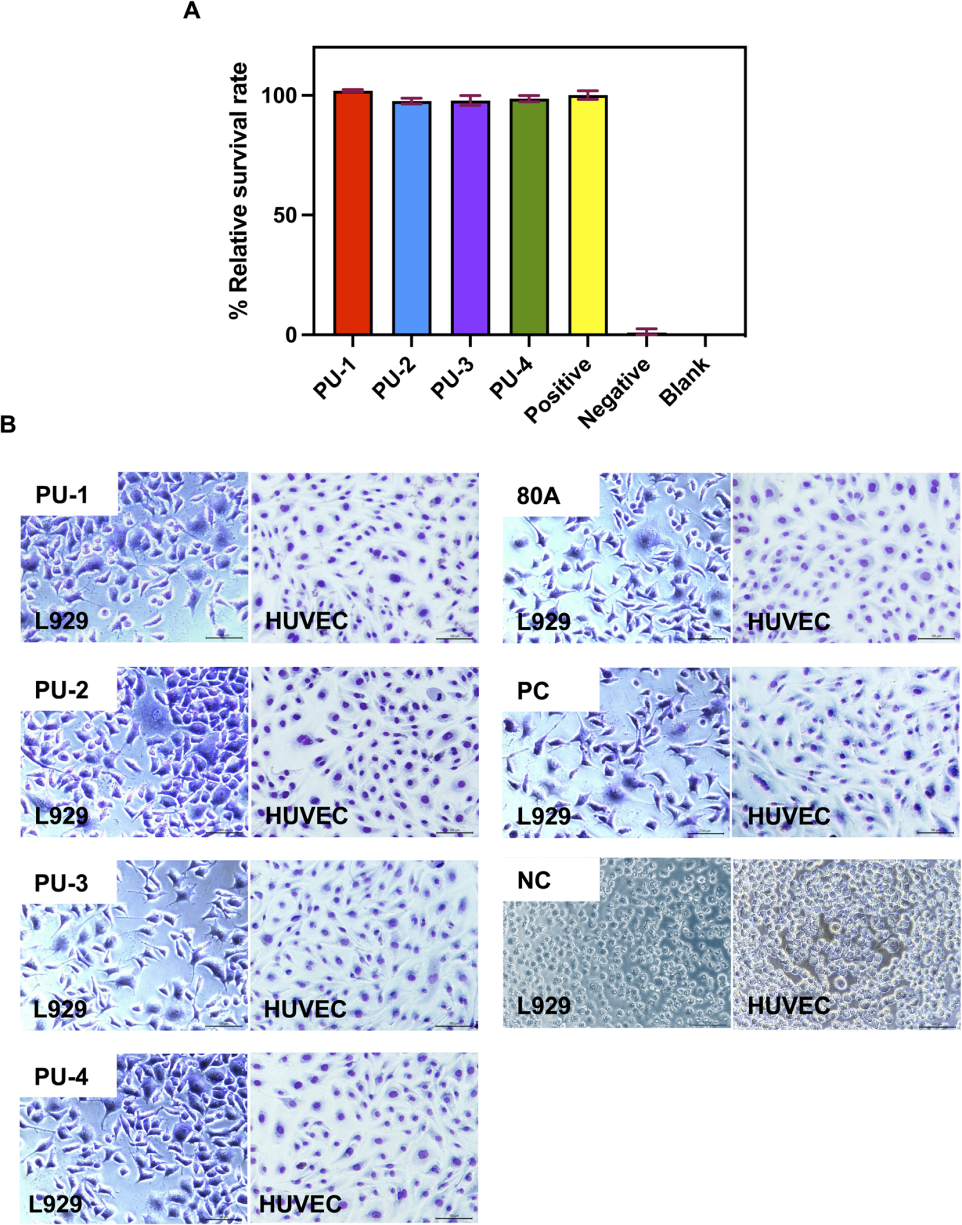
Cell toxicity assays. (A) Cell relative proliferation rates of PUs. (B) Cell morphology of L929 cells and HUVEC cells cultured in the leachate of each material.

We utilized L929 mouse fibroblasts and HUVEC human umbilical vein endothelial cells to assess the cytotoxicity of the tested materials by observing cell morphology and relative growth rate. Fig. 7 displays the cell morphology of the two cell types cultured in the extraction fluids of different polymers. The cells exhibit good growth, regular morphology, and minimal cell death or apoptosis in the extraction fluids.

#### 3.2.2 Macrophages and inflammatory response

**Fig. 8:**
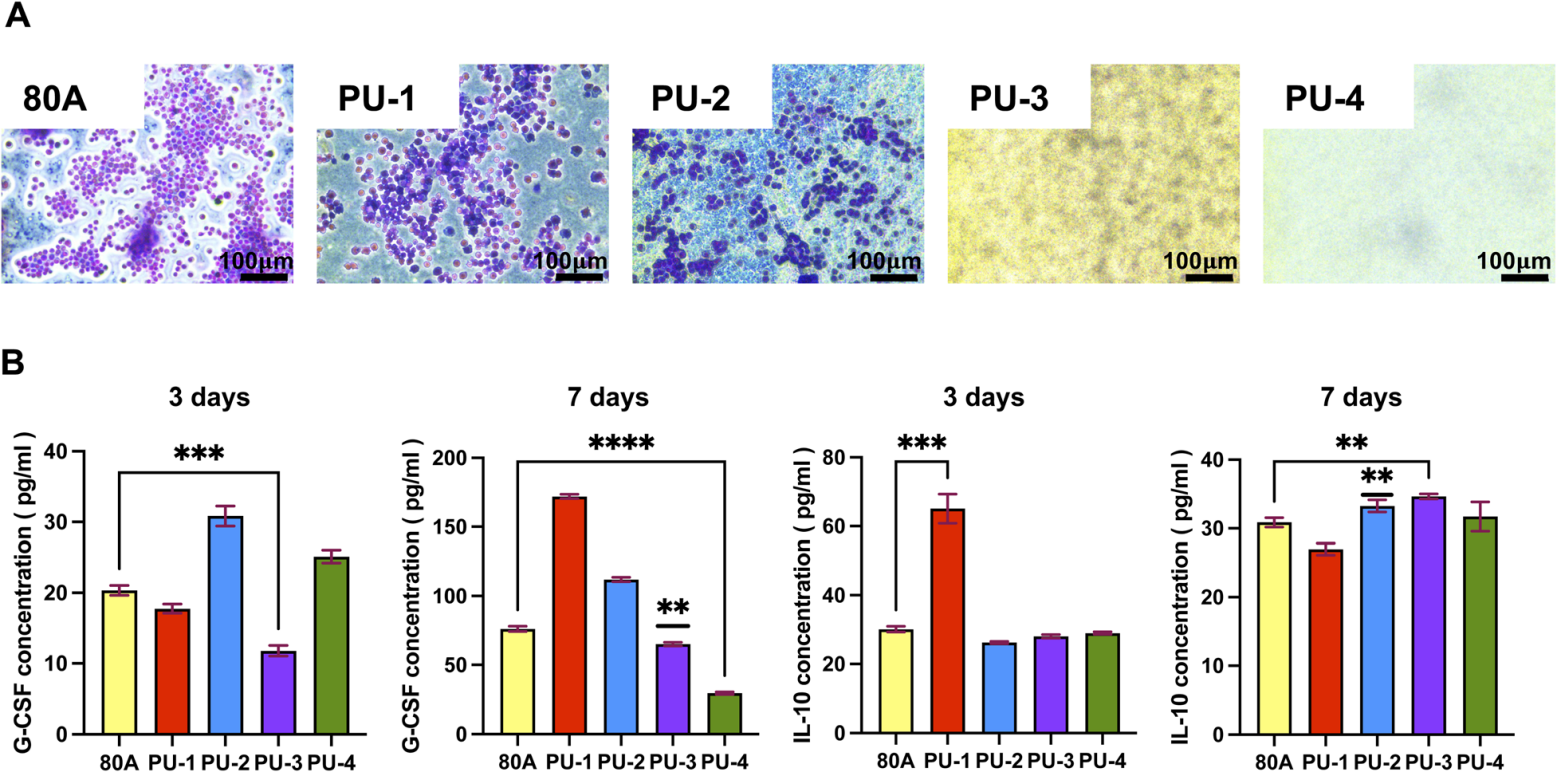
Evaluation of macrophage behavior *In vitro* on material surfaces. (A) Morphology and quantification of macrophages cultured *in vitro* for 3 days. (B) Cytokine expression by macrophages after 3 days and 7 days.

Macrophage recruitment and adhesion play a crucial role in inflammation.^26^ Literature suggests that macrophage adsorption on the material surface can accelerate the degradation of polymeric materials *in vivo*.^27^ Therefore, this study focused on observing and analyzing macrophage adsorption on different material surfaces (Fig. 8). The results indicate that compared to material 80 A, PU-1 and PU-2 adsorb fewer macrophages, while PU-3 and PU-4 surfaces almost have no macrophage adsorption. Additionally, we analyzed the secretion of two cytokines by macrophages. After culturing macrophages on the material surface for 3 days, we observed a significant reduction in the pro-inflammatory factor G-CSF in PU-3 compared to 80 A, suggesting that PU-3 is less likely to induce short-term inflammation. At the same time point, the anti-inflammatory factor IL-10 secreted by macrophages in PU-1 was significantly higher than in 80 A, indicating that PU-1 may have anti-inflammatory potential in the early stages. Further research revealed that after culturing macrophages on the material surface for 7 days, the secretion of pro-inflammatory factors in PU-3 and PU-4 was significantly lower than in 80 A, suggesting that in the long term, PU-3 and PU-4 materials have lower pro-inflammatory reactions than 80 A.

#### 3.2.3 Adhesion and activation of blood cells

**Fig. 9:**
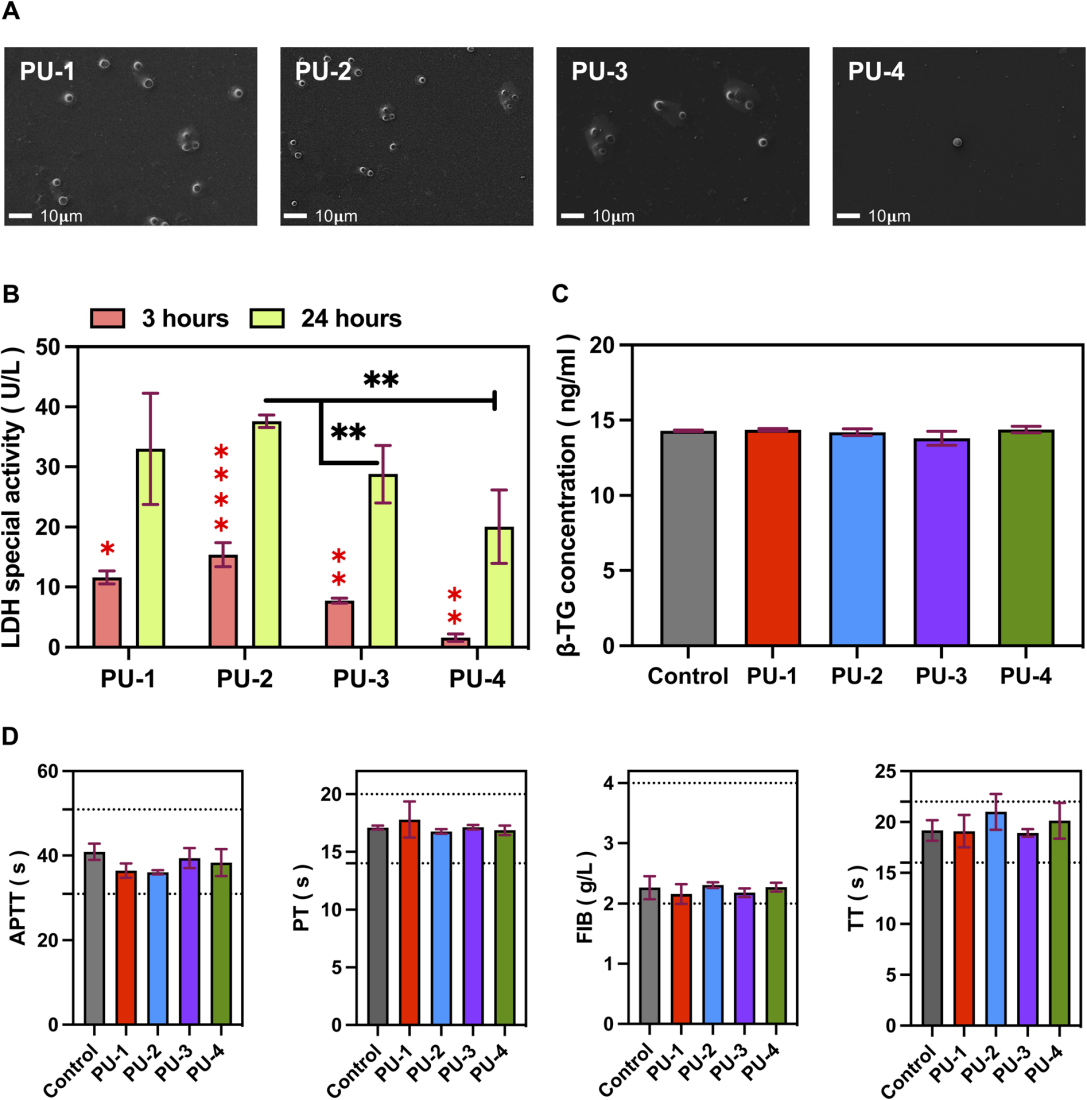
Adhesion and activation of blood cells. (A) Scanning electron microscopy images showing the number and morphology of adherent blood cells on material surfaces, magnified at 500×. (B) Semi-quantitative analysis of blood cell adhesion using lactate dehydrogenase. Red asterisks indicate intra-group differences, while black asterisks indicate inter-group differences. (C) Platelet activation assessed by β-thromboglobulin content. (D) Coagulation parameters, including activated partial thromboplastin time (APTT), fibrinogen (FIB), prothrombin time (PT), and thrombin time (TT).

Adhesion and activation of blood cells on the surface of biomaterials are critical in the field of biomedicine. This process involves interactions between platelets, white blood cells, red blood cells, and the surface of biomaterials. To study the adhesion of blood cells on material surfaces, we used scanning electron microscopy to observe blood cell attachment (Fig. 9). The results show that PU-1 and PU-2 materials adsorb more blood cells, while PU-3 and PU-4 have almost no blood cell adsorption. Furthermore, we used lactate dehydrogenase semi-quantitative analysis to further evaluate blood cell adsorption on the material surface. Compared to incubation for 3 hours, after 24 hours of incubation, the residual content of lactate dehydrogenase on the material surfaces increased. Meanwhile, the residual lactate dehydrogenase content on the surface of PU-4 was the lowest, consistent with electron microscopy observations. Additionally, we further studied the activation of platelets by observing β-TG content. The results show that none of the four materials activated platelets. Finally, we tested the impact of materials on the coagulation system, and the results of the four coagulation indicators suggest that these four materials did not significantly affect the coagulation system, with all coagulation parameters within the normal range.

#### 3.2.4 Protein adsorption test

**Fig. 10:**
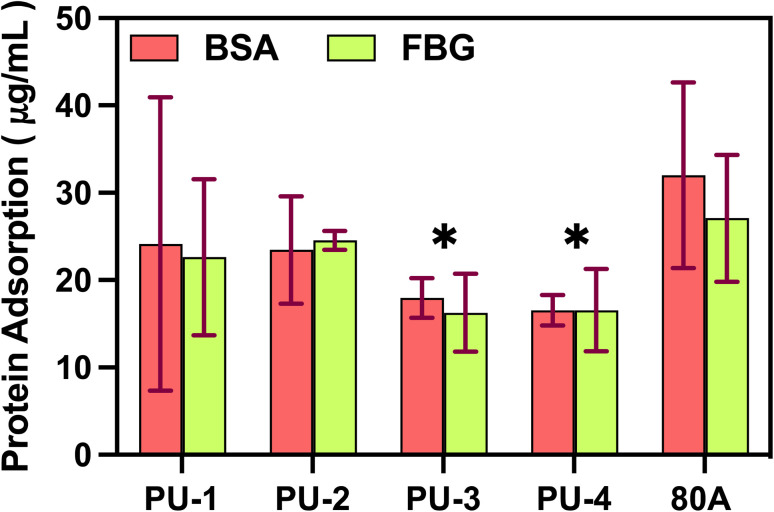
Adsorption of Bovine Serum Albumin (BSA) and fibrinogen (FBG) proteins. * Represents *P*-values less than 0.05, indicating significant differences compared to 80 A for each material.

We conducted an in-depth study on the adsorption properties of material surfaces with two key proteins, bovine serum albumin (BSA) and fibrinogen (FBG). As shown in the Fig. 10, PU-3 and PU-4 exhibit a significant decrease in protein adsorption compared to the surface of 80 A.

#### 3.2.5 Subcutaneous implantation in rats

**Fig. 11:**
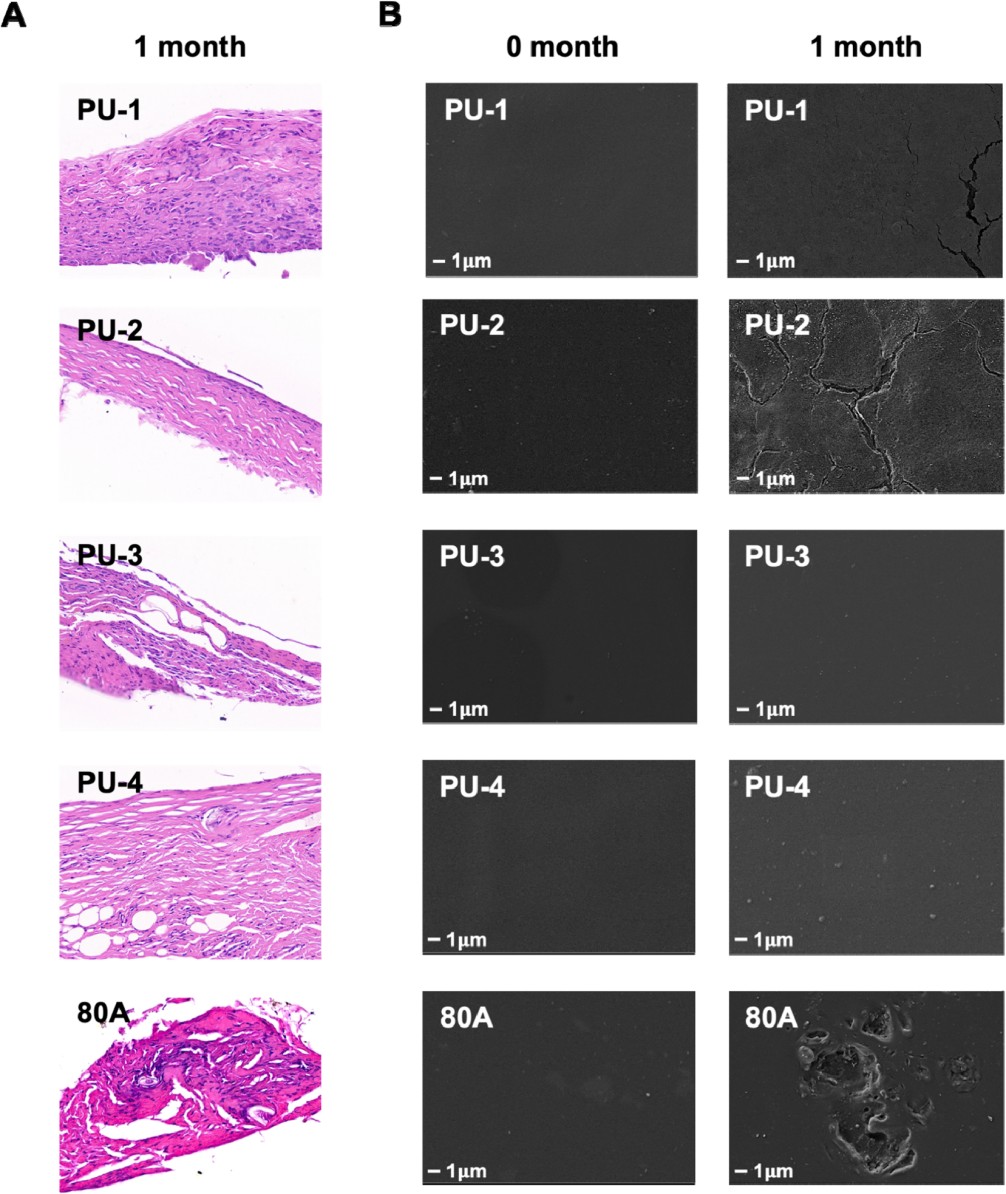
Subcutaneous implantation experiment in rats. (A) Histological examination of tissues surrounding the material 30 days post-implantation using H&E staining. (B) Scanning electron microscopy images depicting the surface condition of the material 30 days post-implantation, magnified at 5000 times.

**Fig. 12:**
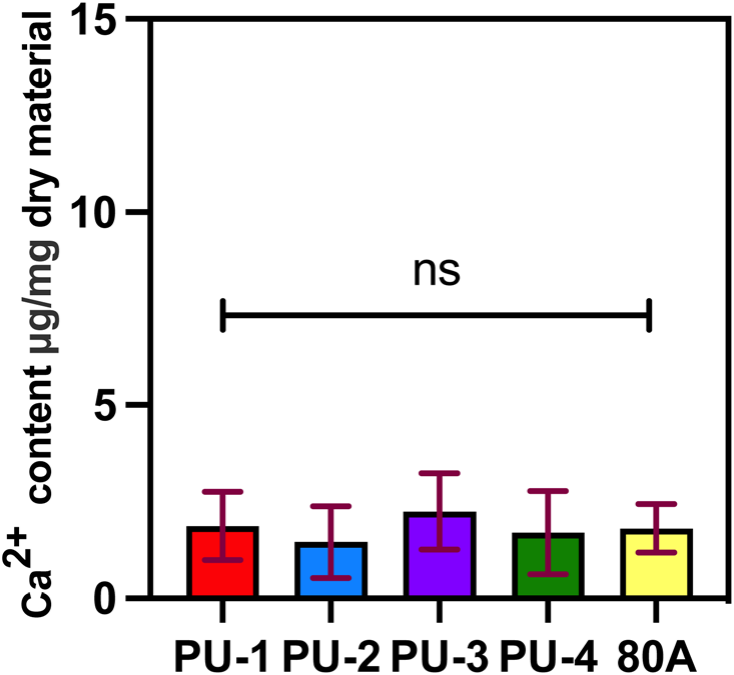
ICP-OES calcium ion content test.

To evaluate the biocompatibility and degradation level of biomaterials *in vivo*, we implanted the materials into the subcutaneous tissue of rats. We found that all five materials were surrounded by connective tissue, with varying degrees of infiltration of inflammatory cells. Compared to 80 A, the infiltration of inflammatory cells was relatively lower for the four materials (Fig. 11). Based on electron microscope results, we found that the surfaces of PU-1, PU-2, and 80 A underwent different degrees of cracking, indicating that these materials may have experienced some degree of degradation due to the invasion of inflammatory cells under physiological conditions. After implantation in rat subcutaneous tissue, PU-3 and PU-4 showed less infiltration of surrounding inflammatory cells, and the material surface remained smooth with minimal signs of degradation, indicating that PU-3 and PU-4 have excellent biocompatibility and biodurability. Based on this, we conducted calcium content determination experiments to detect the calcification process within the implanted materials (Fig. 12). The results revealed that none of the four polymer materials implanted exhibited significant calcification compared to the control group 80 A, showing no significant difference.

## 4 Discussion

### 4.1 Performance characterization

This study comprehensively evaluated the performance of four materials through diverse characterization techniques to investigate the correlation between their structure and properties. Initially, the molecular structure of the materials was preliminarily examined using FTIR spectroscopy. The high sensitivity of FTIR enabled the detection of specific functional group vibrations, providing insights into the basic molecular structure. This initial analysis laid the foundation for understanding the chemical composition of the materials, facilitating subsequent in-depth analyses.

In terms of elemental composition, SEM-EDX and XPS techniques were employed. SEM-EDX provided visual observations of material surface morphology and elemental distribution, while XPS revealed the distribution of elements at different depths. These techniques offered crucial information on the chemical environment at material surfaces and interfaces, as well as the spatial distribution of elements.

Subsequently, the microphase separation performance of the materials was assessed using SAXS and DSC. SAXS's high resolution allowed the observation of changes in the materials' microstructure, contributing to an understanding of their phase separation characteristics. DSC results corroborated the SAXS findings, indicating a theoretical basis for the thermal processing of PU-2 within the 160–190 °C range.

From a thermal stability perspective, TGA analysis provided insights into the materials' performance under high-temperature conditions. Evaluating these thermodynamic properties deepened our understanding of the materials' thermal behavior, offering a theoretical foundation for their processing applications at elevated temperatures.

Finally, detailed studies were conducted on the materials' wettability and mechanical properties. Surface hydrophobicity testing provided insights into the materials' interactions with the external environment. Mechanical property testing revealed key characteristics such as strength and hardness. This comprehensive performance assessment enabled a thorough understanding of various aspects of the materials, laying the groundwork for their practical performance in diverse applications.

In summary, through these comprehensive characterization techniques, we gained in-depth insights into the molecular structure, microphase separation, elemental composition, thermodynamic properties, wettability and mechanical performance of the materials. The exploration of their interrelationships will provide robust guidance for future material design and performance optimization, driving research and applications in related fields.

#### 4.1.1 Structural analysis

In this study, Fourier-transform infrared spectroscopy (FTIR) was employed to analyze the molecular structure of the PUs. The results indicated that all four materials exhibited characteristic absorption peaks related to polyurethane and siloxane, suggesting their composition from these two components. Differences in the distribution of polyurethane characteristic absorption peaks around 1700 cm^−1^ were observed between PU-1 and the others (PU-2, PU-3, and PU-4). This variation may be attributed to PU-1 being a polycarbonate-type polyurethane, while PU-2, PU-3, and PU-4 are polyether-type polyurethanes. These findings provided crucial information about the molecular structure and components of these materials, essential for understanding their performance.

#### 4.1.2 Interface analysis

Combined scanning electron microscopy and energy-dispersive X-ray spectroscopy (SEM-EDX) were utilized to determine the elemental composition of the material surfaces. The siloxane polyurethanes exhibited a significant silicon content, while polyurethane 80 A lacked silicon. This discovery is crucial, as the presence of silicon may impact material performance and biocompatibility. Particularly, the high silicon content in PU-3 and PU-4 might lead to slightly inferior mechanical performance. This outcome provides essential criteria for selecting materials suitable for specific applications.

Building upon the preliminary SEM-EDX analysis of surface elemental composition, XPS was further employed to assess the element distribution at different depths, exploring the evolution of the material's surface chemical environment. The concentration of silicon and oxygen elements gradually decreased with increasing depth. This prompted a deeper consideration of the distribution pattern of silicon elements in siloxane polyurethanes. We speculated that silicon elements in siloxane polyurethanes may preferentially accumulate at the material surface due to their lower free energy, explaining the decrease in silicon concentration with increasing etching depth. Simultaneously, the aggregation of silicon and oxygen elements on the material surface in the form of siloxane suggested a relationship with minimizing surface energy and interacting with adjacent elements.^28^ XPS depth profiling revealed critical features of the material surface's elemental composition, offering valuable information for a profound understanding of material performance and applications.

#### 4.1.3 Microphase separation degree analysis

SAXS analysis of siloxane-containing polyurethane materials revealed significant microphase separation structures, demonstrating the incompatibility between soft and hard segments with an interdomain spacing in the range of 15–30 nm. This microphase separation structure not only provided a structural foundation for the material's excellent mechanical performance but also enhanced its biocompatibility. The results indicated a higher degree of microphase separation in PU-1 and PU-4, with PU-1 exhibiting superior tensile performance, likely a consequence of its pronounced microphase separation. However, the prominent microphase separation degree might adversely affect the creep performance of the materials. Materials with significant microphase separation indicate severe aggregation of the hard segment in polyurethane materials, leading to creep and irreversible deformation during cyclic stretching. This could be a reason for the relatively poor creep performance of PU-1 and PU-4. For polymer heart valve materials, tensile performance and creep are crucial mechanical indicators. Analyzing the material's microphase structure through SAXS to explore the factors influencing mechanics is a crucial testing method, providing technical support and insights for selecting materials with excellent creep and mechanical properties in subsequent studies. DSC results revealed a trend of decreasing and then increasing glass transition temperatures (*T*_g_) for siloxane in PU-1, PU-4, and PU-3. This trend aligned with the SAXS test results. PU-1 displayed two distinct glass transition temperatures in DSC testing, indicating a mixed soft segment with minor differences in content. This consistency with the XPS result, which showed a Si content of 17% in PU-1, supports the interpretation. PU-2, lacking siloxane, exhibited no *T*_g_. This could be due to either a low siloxane content or excellent thermodynamic compatibility between different phases, with the limited siloxane dissolving in other phases and no longer displaying its original thermodynamic characteristics. PU-3 and PU-4 with siloxane showed *T*_g_ because of the higher content of siloxane in the soft segment and sufficient phase separation. This observation aligns with the results obtained from interface analysis and mechanical performance. Additionally, PU-2 exhibited a melting endothermic peak in the range of 160–190 °C in DSC, indicating excellent thermal processing performance.

#### 4.1.4 Thermal stability

Thermal stability analysis of the materials revealed the impact of thermal processing and the addition of siloxane on their stability. The results demonstrated that the addition of siloxane did not alter the thermal stability of the materials. All siloxane polyurethanes exhibited excellent thermal stability, with initial decomposition temperatures around 270 °C and maximum decomposition temperatures exceeding 300 °C. Moreover, PU-2 showed outstanding thermal processing performance, with a melting temperature around 180 °C and a thermal decomposition temperature around 270 °C.

#### 4.1.5 Mechanical properties

Firstly, it is important to recognize the complexity of the location where artificial heart valves are positioned, which determines the specific properties required of their materials. Apart from biocompatibility and bio-stability, mechanical performance is vital for ensuring the long-term stability and reliability of implants. Therefore, in our study, we particularly focused on the mechanical performance of four different materials, including tensile strength, Young's modulus, elongation at break, and creep performance. These data are crucial for selecting materials suitable for heart valve applications as they must withstand the cyclical motion and high-pressure environment within the heart.

Our research findings indicate that compared to the silicon-based polyurethane material LifePolymer™ (tensile strength 35 MPa, Young's modulus 20 MPa, elongation at break 681%) used in polymer valve prostheses already undergoing clinical trials by Foldax company,^9^ our materials PU-1 and PU-2 exhibited tensile strengths of 36.31 MPa and 37.67 MPa, respectively, significantly higher than those of Foldax's product. However, the mechanical performance of PU-3 and PU-4 was relatively lower but still around 30 MPa.

One possible reason for the difference in tensile strength is the varying silicone content in the materials. Silicone polymers have lower intermolecular forces, so an excessive amount of silicone can reduce the mechanical strength of the material, consistent with our surface analysis results. Additionally, an excess of silicone may reduce material compatibility, thereby affecting its phase separation and, consequently,^29^ its mechanical performance.

The importance of elastic modulus cannot be overstated in the selection of heart valve materials as it directly affects hemodynamics. A higher elastic modulus may result in a smaller valve opening area, increasing the burden on the patient's heart. According to literature reports, the elastic modulus of materials used for artificial heart valves should be less than 35 Mpa,^9^ a requirement met by all four materials in our study.

Furthermore, due to the continuous stress exerted on heart valves in their working environment, creep fatigue leading to fracture is one of the primary failure modes of polymer materials. Creep rate is an important indicator of a material's resistance to creep, representing the slope of the curve during continuous stress loading to unloading. Our study revealed that PU-1, PU-3, and PU-4 exhibited extremely low creep rates, while PU-2 demonstrated poorer resistance to creep. Under a continuous stress of 4 MPa for one hour, the irreversible deformation was less than 10%, meeting the requirements for heart valve materials. Particularly, PU-3 showed an exceptionally low creep rate and irreversible deformation, indicating optimal creep performance.

Considering the results of tensile strength, elastic modulus, and creep performance tests, PU-3 demonstrated excellent characteristics in mechanical performance, making it potentially more suitable for heart valve applications.

#### 4.1.6 Wettability and water absorption

The combined analysis of wettability and water absorption revealed the potential applications of the four polymer materials in the biomedical field. Hydrophobic surfaces are advantageous in reducing adhesion of blood and other biological fluids,^30^ slowing down the degradation process within the body. Low water absorption rates indicate material stability in humid environments, supporting prolonged use in conditions such as heart valves. The joint assessment of these properties provides a basis for considering these materials in biomedical applications.

### 4.2 Biocompatibility

#### 4.2.1 Cytotoxicity

Biocompatibility is a critical property of medical biomaterials. In our cytotoxicity tests, leachates from the four materials exhibited low cytotoxicity towards mouse fibroblasts and human umbilical vein endothelial cells. This implies that these materials are unlikely to have harmful effects on cells, a positive characteristic for their biomedical applications.

#### 4.2.2 Inflammatory response and macrophage interaction

To investigate the interaction between materials and macrophages and the consequent immune response, we utilized two inflammatory cytokines, G-CSF and IL-10, as indicators of the extent of inflammation induced by the materials. G-CSF, a pro-inflammatory factor, plays a crucial role in the inflammatory response.^31^ On the other hand, IL-10 acts as an anti-inflammatory factor, regulating inflammation.^32^

Our experimental results revealed that after 7 days of contact between the materials and macrophages, the concentration of G-CSF in the leachate of PU-3 and PU-4 was significantly lower than that in the leachate of the control group, 80 A. The decreased levels of G-CSF indicate that compared to 80 A, the materials exhibit weaker pro-inflammatory capabilities. This observation could be attributed to the higher silicon content in PU-3 and PU-4 materials, which forms a silicon protective layer on the material surface, hindering macrophage adhesion. As a result, macrophages are less likely to adhere to the material surface, thus reducing the triggering of immune responses and inflammation upon material implantation.

Furthermore, after 7 days of material implantation, the concentration of IL-10 in the leachate of PU-3 was significantly higher than that in the leachate of 80 A. IL-10, being an anti-inflammatory factor, its lower concentration in the case of 80 A may not be sufficient to counteract the occurrence of inflammation. Therefore, compared to 80 A, PU-3 demonstrates superior inflammatory response capabilities, which is significant in the context of material biocompatibility and immunogenicity assessment.

In summary, our findings suggest that PU-3 and PU-4 materials exhibit lower pro-inflammatory potential compared to 80 A due to their higher silicon content, which inhibits macrophage adhesion and subsequent immune responses. Additionally, the elevated IL-10 levels observed in PU-3 indicate its potential as a favorable material in terms of inflammatory response regulation. These insights contribute to our understanding of material–host interactions and have implications for biomedical applications.

#### 4.2.3 Adhesion and activation of blood cells

The adhesion and activation of blood cells on the surface of biomaterials are important considerations. Our research found that PU-3 and PU-4 exhibited very low adhesion of blood cells and did not induce platelet activation. This is crucial for preventing thrombus formation and excessive inflammatory reactions. Additionally, coagulation system tests indicated that these materials had no significant impact on the clotting system, further confirming their biocompatibility.

#### 4.2.4 Protein adsorption

This study preliminarily explored the interaction between proteins and the surface of artificial valve materials, a process crucial for the biocompatibility and performance stability of artificial valves in the body. The adsorption behavior of proteins on the surface of artificial valves not only affects the surface properties of the materials but also regulates the body's response to the artificial valves.^33^ Excessive protein adsorption in medical device applications can lead to various issues.^34^ PU-3 and PU-4 materials demonstrated weak protein adsorption, likely attributed to their higher silicon element content. The introduction of silicon elements may adjust the surface charge distribution,^35^ affecting the interaction between proteins and materials. The high electronegativity of silicon may lead to the formation of a charge shielding layer on the surface, slowing down the adsorption of bovine serum albumin (BSA) and fibrinogen (FBG) on PU-3 and PU-4. This charge shielding effect is expected to reduce the affinity between proteins and the material surface, lowering the adsorption capacity. This finding not only provides profound insights into our understanding of the protein-material interaction mechanism but also offers valuable guidance for designing artificial valve materials with enhanced biocompatibility and anti-thrombotic properties.

#### 4.2.5 Subcutaneous implantation in rats

In the subcutaneous implantation experiment in rats, we observed the formation of connective tissue around all materials, but there was minimal infiltration of inflammatory cells on the material surfaces, and PU-3 and PU-4 surfaces showed almost no signs of degradation. This indicates good biocompatibility and resistance to degradation, making them suitable for long-term implantation applications.

In this study, we characterized the level of calcification by measuring the calcium content using ICP-OES testing after 30 days of material implantation subcutaneously in rats. The results indicate that none of the four materials showed significant calcification after implantation, and there was no significant difference compared to the control group 80 A. In previous literature, it has been reported that the calcium content of bioprosthetic valves fixed with glutaraldehyde and implanted subcutaneously in rats for 30 days was measured at 100.01 ± 7.13 micrograms per milligram *via* ICP-OES analysis.^36^ In contrast, the calcium content of the four polymer materials currently under our investigation is significantly lower. This finding further highlights the superiority of polymer materials over bioprosthetic valves in terms of anti-calcification properties. It indicates that our materials possess better anti-calcification capabilities, which are crucial for long-term implantable cardiac valve materials. This result provides robust support for our study and underscores the potential application value of polymer materials in the field of cardiac valve prostheses.

It is worth noting that PU-3 and PU-4, as polymers with high silicon content, exhibited excellent surface characteristics with low cell adhesion. This is attributed to the presence of silicon elements with a lower surface energy, which may alter the elemental distribution on the material surface, making it less prone to cell adhesion. This characteristic is significant in biomedical applications as it effectively reduces potential inflammatory cell adhesion, lowers the risk of inflammatory reactions, and also reduces blood cell adhesion, thus minimizing the potential danger of thrombus formation. Moreover, this feature contributes to enhancing the material's biocompatibility in specific applications. Based on these advantages, PU-3 and PU-4 have the potential for widespread applications, especially in the field of artificial heart valves, making them promising high-molecular-weight polymer materials. This not only positively influences the improvement of the performance and reliability of medical materials but also provides a valuable template for future biomedical material design.

In conclusion, our study provides a comprehensive assessment of the silicon-containing polyurethane materials in terms of performance characterization and biocompatibility. The results offer strong support for the selection of materials suitable for heart valve applications and provide useful references for future biomedical material design. However, further research is needed to validate these results and gain a deeper understanding of the performance of these materials in different clinical applications.

## 5 Conclusions

Through a series of tests, we conducted a detailed analysis of the performance of silicone-oxygen polyurethane in mechanical properties, biocompatibility, and biological stability. The results demonstrate outstanding mechanical performance for PU-1 and PU-2, with a tensile strength of 36 MPa for PU-1 and 38 MPa for PU-2. Meanwhile, PU-3 and PU-4 exhibited superior biological stability and biocompatibility.

By combining SEM-EDX and XPS analyses, we explained the surface migration phenomenon of silicon elements in silicone-oxygen polyurethane. This surface migration is a key factor contributing to the enhanced biocompatibility and biological stability compared to 80 A. The surface migration of silicon elements may form a “silicon protective layer”, reducing the material's surface affinity, thereby slowing down cell and protein adsorption and enhancing biocompatibility. Additionally, surface elemental analysis revealed that the high silicon content in PU-3 and PU-4 is a major factor causing the reduction in mechanical performance while significantly improving biocompatibility and biological stability.

Considering the comprehensive factors of mechanical properties, biocompatibility, and biological stability, the high silicon content in PU-3 positions it with extensive prospects in biomedical applications. This study provides profound insights into the selection of high-molecular-weight polymer materials for artificial heart valve applications and emphasizes the importance of considering different performance indicators in material design. The superior performance of PU-3 makes it a promising candidate material worthy of in-depth research and development in the field of artificial heart valves in the future.

## Conflicts of interest

There are no conflicts to declare.

## Supplementary Material

## References

[cit1] German K. S., Kalra A. (2019). Curr. Opin. Support. Palliat. Care.

[cit2] Aluru J. S., Barsouk A., Saginala K., Rawla P., Barsouk A. (2022). Med. Sci..

[cit3] Yu J., Qiao E., Wang W. (2022). Clin. Cardiol..

[cit4] Leviner D. B., Zafrir B., Saliba W., Stein N., Shiran A., Sharoni E. (2022). Eur. J. Cardio. Thorac. Surg..

[cit5] Sanaani A., Yandrapalli S., Harburger J. M. (2018). Cardiol. Rev..

[cit6] Gopal S. , Hauser J. M. and Mahboobi S. K., in StatPearls, StatPearls Publishing Copyright © 2023, StatPearls Publishing LLC., Treasure Island (FL), 2023

[cit7] Durand E., Sokoloff A., Urena-Alcazar M., Chevalier B., Chassaing S., Didier R., Tron C., Litzler P. Y., Bouleti C., Himbert D., Hovasse T., Bar O., Avinée G., Iung B., Blanchard D., Gilard M., Cribier A., Lefevre T., Eltchaninoff H. (2019). Circ.: Cardiovasc. Interventions.

[cit8] Purinya B., Kasyanov V., Volkolakov J., Latsis R., Tetere G. (1994). J. Biomech..

[cit9] Dandeniyage L. S., Gunatillake P. A., Adhikari R., Bown M., Shanks R., Adhikari B. (2018). J. Biomed. Mater. Res., Part B.

[cit10] Dandeniyage L. S., Adhikari R., Bown M., Shanks R., Adhikari B., Easton C. D., Gengenbach T. R., Cookson D., Gunatillake P. A. (2019). J. Biomed. Mater. Res., Part B.

[cit11] Gallagher G., Padsalgikar A., Tkatchouk E., Jenney C., Iacob C., Runt J. (2017). J. Biomed. Mater. Res., Part B.

[cit12] Jenney C., Millson P., Grainger D. W., Grubbs R., Gunatillake P., McCarthy S. J., Runt J., Beith J. (2020). Adv. NanoBiomed Res..

[cit13] Li R. L., Russ J., Paschalides C., Ferrari G., Waisman H., Kysar J. W., Kalfa D. (2019). Biomaterials.

[cit14] Kaplan J., Grinstaff M. (2015). J. Visualized Exp..

[cit15] Thomas V., Jayabalan M. (2009). J. Biomed. Mater. Res., Part A.

[cit16] Rudolph A., Teske M., Illner S., Kiefel V., Sternberg K., Grabow N., Wree A., Hovakimyan M. (2015). PLoS One.

[cit17] Celesti C., Iannazzo D., Espro C., Visco A., Legnani L., Veltri L., Visalli G., Di Pietro A., Bottino P., Chiacchio M. A. (2022). Materials.

[cit18] Tang L., Long X., He X., Ding M., Zhao D., Luo F., Li J., Li Z., Tan H., Zhang H. (2021). J. Mater. Chem. B.

[cit19] D'Amore A., Luketich S. K., Raffa G. M., Olia S., Menallo G., Mazzola A., D'Accardi F., Grunberg T., Gu X., Pilato M., Kameneva M. V., Badhwar V., Wagner W. R. (2018). Biomaterials.

[cit20] Kidane A. G., Burriesci G., Edirisinghe M., Ghanbari H., Bonhoeffer P., Seifalian A. M. (2009). Acta Biomater..

[cit21] Singh S. K., Kachel M., Castillero E., Xue Y., Kalfa D., Ferrari G., George I. (2023). Front. Cardiovasc. Med..

[cit22] Spigel Z. A., Zhu H., Qureshi A. M., Penny D. J., Caldarone C. A., Heinle J. S., Binsalamah Z. M. (2021). Semin. Thorac. Cardiovasc. Surg..

[cit23] Khan I., Smith N., Jones E., Finch D. S., Cameron R. E. (2005). Biomaterials.

[cit24] Simmons A., Hyvarinen J., Poole-Warren L. (2006). Biomaterials.

[cit25] Ishihara H., Kimura I., Saito K., Ono H. (1974). J. Macromol. Sci., Part B.

[cit26] Yunna C., Mengru H., Lei W., Weidong C. (2020). Eur. J. Pharmacol..

[cit27] Yahyouche A., Zhidao X., Czernuszka J. T., Clover A. J. (2011). Acta Biomater..

[cit28] Liu Z.-h., Xiao Y.-h., Ma X.-y., Geng X., Ye L., Zhang A.-y., Feng Z.-g. (2022). Mater. Adv..

[cit29] Wang W., Bai X., Sun S., Gao Y., Li F., Hu S. (2022). Int. J. Mol. Sci..

[cit30] Zhong L., Jin J., Zheng D., Guan W., Guo Y., Chen A., Peng Y., Gao Q., Zheng Y., Huang H. (2018). J. Mater. Sci.: Mater. Med..

[cit31] Venet F., Monneret G. (2018). Nat. Rev. Nephrol..

[cit32] Nagata K., Nishiyama C. (2021). Int. J. Mol. Sci..

[cit33] Buck E., Lee S., Gao Q., Tran S. D., Tamimi F., Stone L. S., Cerruti M. (2022). ACS Biomater. Sci. Eng..

[cit34] Xu L. C., Siedlecki C. A. (2017). J. Biomed. Mater. Res., Part B.

[cit35] Lin K., Li Z., Tao Y., Li K., Yang H., Ma J., Li T., Sha J., Chen Y. (2021). Langmuir.

[cit36] Hu M., Peng X., Shi S., Wan C., Cheng C., Lei N., Yu X. (2022). J. Mater. Chem. B.

